# Atypical Trigger Finger Associated With Proximal Flexor Digitorum Superficialis Dynamic Instability Diagnosed With Ultrasound and Treated With Hydrodissection: A Case Report

**DOI:** 10.7759/cureus.113614

**Published:** 2026-07-29

**Authors:** Yonghyun Yoon, King Hei Stanley Lam, Teinny Suryadi, Anwar Suhaimi, Daniel Chiung-Jui Su

**Affiliations:** 1 Orthopedics, International Academy of Musculoskeletal Medicine, Hong Kong, HKG; 2 Orthopedics, International Academy of Regenerative Medicine, Incheon, KOR; 3 Orthopedics, MSKUS, San Diego, USA; 4 Orthopedic Surgery, Hallym University Gangnam Sacred Heart Hospital, Seoul, KOR; 5 Orthopedic Surgery, Incheon Terminal Orthopedic Surgery Clinic, Incheon, KOR; 6 Clinical Research, International Academy of Regenerative Medicine, Incheon, KOR; 7 The Board of Clinical Research, The Indonesian Malaysian Regenerative Institute, Jakarta, IDN; 8 The Board of Clinical Research, The International Association of Musculoskeletal Medicine, Kowloon, HKG; 9 Faculty of Medicine, The Chinese University of Hong Kong, New Territories, HKG; 10 Faculty of Medicine, The University of Hong Kong, Hong Kong, HKG; 11 The Board of Clinical Research, The Hong Kong Institute of Musculoskeletal Medicine, Kowloon, HKG; 12 Department of Physical Medicine and Rehabilitation, Medistra Hospital, Jakarta, IDN; 13 Physical Medicine and Rehabilitation, Synergy Clinic, Jakarta, IDN; 14 Department of Physical Medicine and Rehabilitation, Hermina Podomoro Hospital, Jakarta, IDN; 15 Rehabilitation Medicine, University Malaya Medical Centre, Kuala Lumpur, MYS; 16 Rehabilitation Medicine, University Malaya, Kuala Lumpur, MYS; 17 Department of Physical Medicine and Rehabilitation, Chi Mei Medical Center, Tainan, TWN

**Keywords:** dynamic ultrasound, flexor digitorum superficialis, flexor tendon, hydrodissection, medial elbow, occupational disease, stenosing tenosynovitis, tendon instability, trigger finger, ultrasonography

## Abstract

Trigger finger is classically attributed to stenosing pathology at the first annular pulley, and the standard diagnostic and therapeutic approach is largely based on that model. However, some patients present with typical triggering symptoms despite normal distal pulley findings on physical examination and static ultrasonography. We report the case of a 25-year-old female cook with painful triggering of the left third and fourth digits. Examination demonstrated reproducible digital catching without focal tenderness over the involved A1 pulleys, whereas focal tenderness was present at the medial elbow near the proximal flexor-pronator region. Plain radiographs were unremarkable. Static ultrasound of the hand showed normal A1 pulleys and no evidence of tendon nodularity or tenosynovitis. Because of the discordance between symptoms and distal imaging, a systematic dynamic ultrasound examination was extended proximally from the hand to the forearm and medial elbow. This examination demonstrated abnormal motion involving the flexor digitorum superficialis near its proximal origin, with a reproducible snap or shift that coincided with the patient's triggering sensation. Ultrasound-guided hydrodissection with 20 mL of 5% dextrose in water was then performed at the site of the dynamic abnormality. Immediate postprocedure dynamic imaging showed smoother tendon excursion and the absence of the previously observed snap or shift, accompanied by prompt symptomatic improvement. At six weeks, the patient had complete resolution of pain, stiffness, and triggering; had returned to full work duties and recreational rock climbing; and repeat dynamic ultrasound showed persistently normalized tendon motion. This case suggests that, in selected patients with clinically typical trigger finger but normal A1 pulley findings, the symptomatic abnormality may be proximal and dynamic rather than distal and stenotic. Dynamic ultrasound may therefore be useful for evaluating atypical trigger finger presentations and guiding targeted, minimally invasive treatment.

## Introduction

Trigger finger is a common cause of hand pain and functional limitation and is usually explained by impaired gliding of the flexor tendon beneath a diseased A1 pulley [1-3]. Mechanically, triggering is generally thought to occur when narrowing or thickening of the A1 pulley creates a size mismatch between the flexor tendon complex and the pulley, causing the tendon to catch rather than glide smoothly during finger motion. The conventional model emphasizes pulley thickening, tendon enlargement, tenosynovitis, and mechanical mismatch at the metacarpophalangeal level, and this framework underlies the usual treatment algorithms, ranging from splinting and hand therapy to corticosteroid injection and A1 pulley release [3-9].

Ultrasonography has become an important adjunct in the evaluation of trigger finger because it can identify A1 pulley thickening, flexor tendon abnormalities, tenosynovial fluid, and Doppler hypervascularity [10-14]. More recently, dynamic ultrasonography has broadened the assessment beyond static morphology by allowing real-time visualization of tendon motion during symptomatic movement [10,11]. Dynamic sonographic work has also suggested that altered tendon gliding and adherence-related abnormalities may contribute to trigger finger symptoms in at least some patients [15].

Despite this progress, some patients remain diagnostically challenging. Younger adults with repetitive occupational hand use may present with typical triggering symptoms but lack the expected distal sonographic findings [2,10,13]. In such cases, the relevant abnormality may occasionally lie outside the usual pulley-centered field of view. A recent case report described triggering caused by distal flexor tendinopathy rather than A1 pulley disease, further supporting the possibility of uncommon non-pulley mechanisms [16].

Hydrodissection has emerged as a minimally invasive technique for separating restricted soft tissue planes in selected peripheral nerve and tendon-related disorders [17-19]. We report a case of clinically typical trigger finger in a young manual worker whose static hand ultrasound findings were normal but in whom systematic dynamic ultrasound extending proximally identified an abnormal flexor digitorum superficialis motion pattern near the medial elbow. The patient improved after targeted hydrodissection at that site.

## Case presentation

A 25-year-old right-handed female cook presented with a one-year history of painful triggering of the left third and fourth digits, with marked worsening during the week before presentation. She described severe morning stiffness and intermittent locking that partially improved after several minutes of use but became substantially worse with prolonged occupational activity. Her work required repetitive chopping, kneading, gripping, and frequent lifting of heavy cookware for approximately 12 hours per day, six days per week. She had taken over-the-counter nonsteroidal anti-inflammatory medication for two weeks with minimal benefit and had not previously undergone injection therapy or formal rehabilitation. Her medical history was unremarkable. She had no history of diabetes mellitus, thyroid disease, inflammatory arthritis, or prior upper-extremity trauma. She took no regular medications and had no known drug allergies.

Physical examination showed no visible swelling, erythema, deformity, or muscle atrophy in the left hand, wrist, or elbow. Active flexion and extension of the left third and fourth digits reproduced the patient's characteristic catching sensation, with a faint palpable click. Passive motion was smooth and unrestricted. There was no discrete tenderness over the A1 pulleys of the involved digits. In contrast, focal tenderness was present over the medial elbow in the proximal flexor-pronator region near the flexor digitorum superficialis origin, and palpation at this site reproduced the patient's sense of tightness and discomfort. Wrist and elbow range of motion were full. Tinel's sign and Phalen's test at the wrist were negative. Resisted wrist flexion and medial epicondylar stress testing did not meaningfully increase the triggering sensation. Independent flexor digitorum profundus function was preserved. Flexor digitorum superficialis motor function was intact on manual testing; however, the patient's characteristic catching was reproduced during active motion. Flexor tendon function was assessed using standard examination principles for the flexor digitorum profundus and flexor digitorum superficialis [20].

Given the focal medial elbow tenderness, the differential diagnosis included medial epicondylitis, proximal flexor tendinopathy, cubital tunnel syndrome, and other proximal dynamic causes of symptomatic snapping or catching. Medial epicondylitis was considered less likely because resisted wrist flexion and medial epicondylar stress testing did not meaningfully reproduce the patient's characteristic triggering. Cubital tunnel syndrome was considered less likely in the absence of paresthesia, weakness, or a positive Tinel's sign at the cubital tunnel.

Because the symptomatic region was near the cubital tunnel, the ulnar nerve was also assessed at the elbow. Physical examination did not suggest ulnar neuropathy or ulnar nerve instability, and no snapping of the ulnar nerve was detected. Ultrasonographic assessment of the ulnar nerve in the medial elbow region showed no focal fascicular enlargement or diffuse nerve enlargement.

Plain radiographs of the left hand and elbow showed no fracture, degenerative change, joint-space narrowing, or other osseous abnormality. Ultrasonographic evaluation was performed using an Alpinion XC90 Elite ultrasound system (ALPINION MEDICAL SYSTEMS Co., Ltd., Seoul, Republic of Korea) with a linear transducer (center frequency, 7 MHz; operating range, 3-19 MHz). The examination and ultrasound-guided intervention were performed by an orthopedic surgeon with more than 10 years of experience in musculoskeletal ultrasound diagnosis and ultrasound-guided procedures. Static examination of the volar aspect of the third and fourth metacarpophalangeal joints demonstrated normal-appearing A1 pulleys, each measuring <0.5 mm in thickness, without tendon thickening, focal nodules, tenosynovial effusion, or hypervascularity. These findings differed from the typical sonographic pattern reported in conventional trigger finger, in which A1 pulley thickening and tendon-related changes are frequently identified [12-14]. Static sonographic assessment of the medial elbow and proximal forearm showed no clear structural abnormality at the common flexor origin or along the visualized proximal flexor tendons.

Because the patient's symptoms were typical of trigger finger but distal static findings were normal, a systematic dynamic ultrasound examination was then performed, following the flexor tendon system proximally from the hand toward the wrist, forearm, and medial elbow during active finger motion. At the metacarpophalangeal level, dynamic imaging showed mildly altered tendon excursion but no definite impingement at the A1 pulley. The examination was therefore extended to the proximal forearm and medial elbow with the patient supine, the shoulder abducted and externally rotated, the elbow extended, and the forearm supinated.

**Figure 1:**
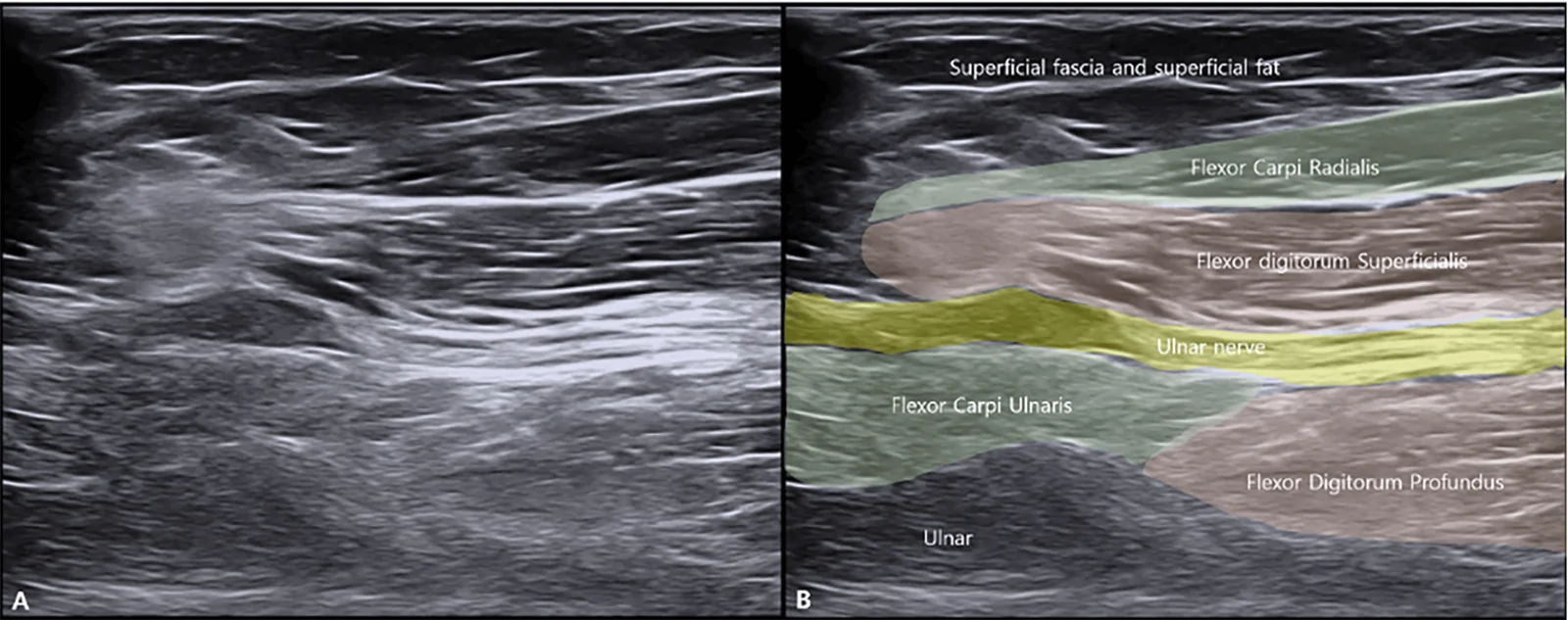
Aberrant gliding of the FDS during dynamic long-axis ultrasound Long-axis ultrasound view of the FDS in the proximal forearm. At the indicated site, approximately 3-4 cm distal to the medial epicondyle, tissue associated with the FDS demonstrated irregular, jerky motion during active finger flexion and extension. This motion abnormality was temporally associated with the patient's triggering sensation and supported the presence of a proximal symptomatic site. FDS, flexor digitorum superficialis Image courtesy: Yonghyun Yoon and King Hei Stanley Lam

Dynamic long-axis imaging near the proximal forearm demonstrated smooth gliding of the flexor digitorum profundus but irregular, jerky motion involving tissue associated with the flexor digitorum superficialis near the proximal myotendinous region (Figure 1, Video 1). The dynamic abnormality was identified qualitatively on the basis of reproducible, symptom-correlated irregular motion observed during repeated active finger flexion-extension cycles; no validated quantitative sonographic metric was available for this specific proximal finding.

**Video 1:** Real-time dynamic long-axis ultrasound demonstrating aberrant FDS gliding This long-axis dynamic ultrasound video shows irregular, jerky motion involving the FDS region in the proximal forearm during active finger motion, approximately 3-4 cm distal to the medial epicondyle. The observed motion abnormality was reproducibly associated with the patient's report of triggering and prompted further focused evaluation of the proximal flexor pathway. FDS, flexor digitorum superficialis

**Figure 2:**
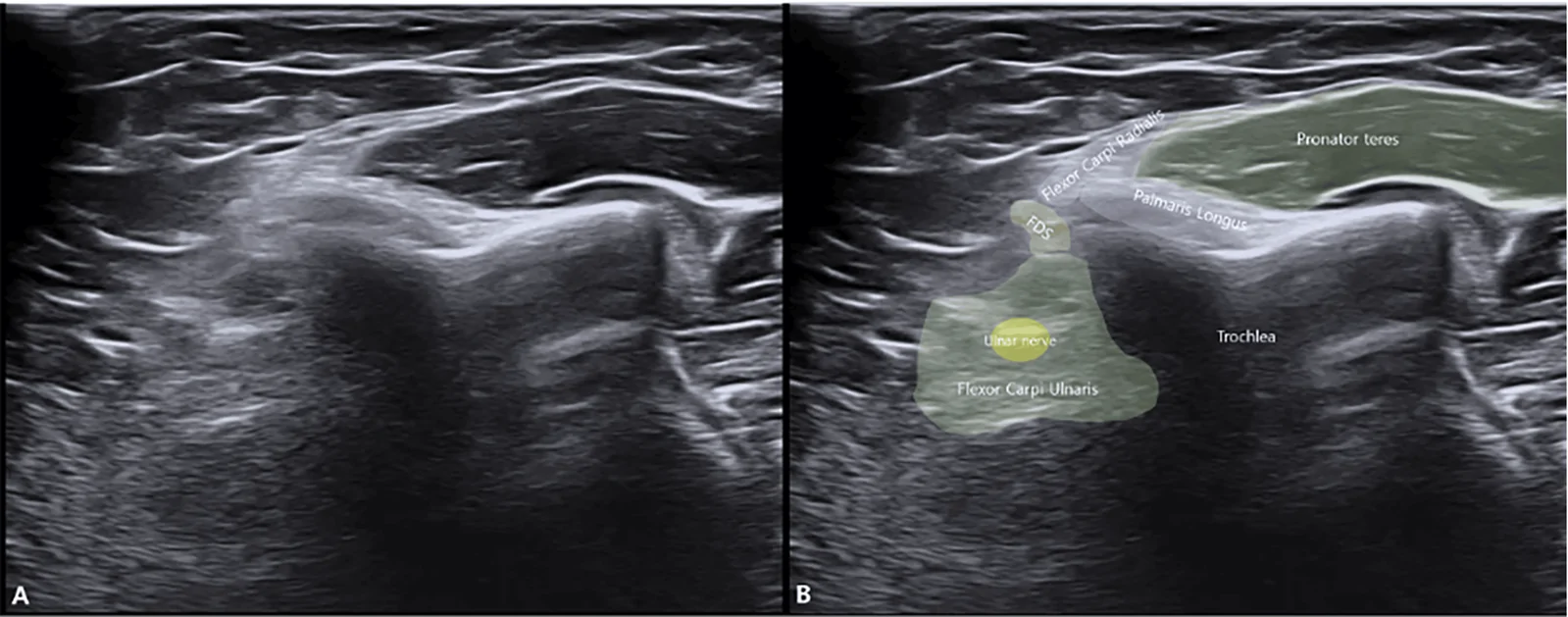
Short-axis dynamic ultrasound showing a symptom-correlated snap or shift in the proximal FDS region Short-axis ultrasound image obtained near the medial elbow at the symptomatic region, approximately 1 cm distal to the proximal FDS origin. During active finger motion, a reproducible snap or shift involving tissue within or immediately adjacent to the proximal FDS was observed at this site. This finding was temporally associated with the patient's characteristic triggering sensation and is presented as a proposed pathoanatomical explanation rather than a definitive diagnosis. FDS, flexor digitorum superficialis Image courtesy: Yonghyun Yoon and King Hei Stanley Lam

Focused short-axis dynamic imaging over the medial elbow then demonstrated a reproducible snap or shift within or immediately adjacent to the proximal flexor digitorum superficialis approximately 1 cm distal to its origin (Figure 2 and Video 2). The patient reported that the same abnormal motion coincided precisely with the sensation she recognized as digital triggering. Although this pattern is not part of the classic distal trigger finger model, it is consistent with the broader concept that altered gliding and adherence-related motion abnormalities may contribute to triggering symptoms [10,11,15].

**Video 2:** Real-time dynamic short-axis ultrasound showing a symptom-correlated snap or shift in the proximal FDS region This short-axis dynamic ultrasound video demonstrates a reproducible snap or shift involving tissue within or immediately adjacent to the proximal FDS during active finger motion. The event was temporally associated with the patient's report of triggering. The finding is interpreted as a symptom-correlated dynamic abnormality and a proposed pathoanatomical explanation rather than definitive proof of causation. FDS, flexor digitorum superficialis

**Figure 3:**
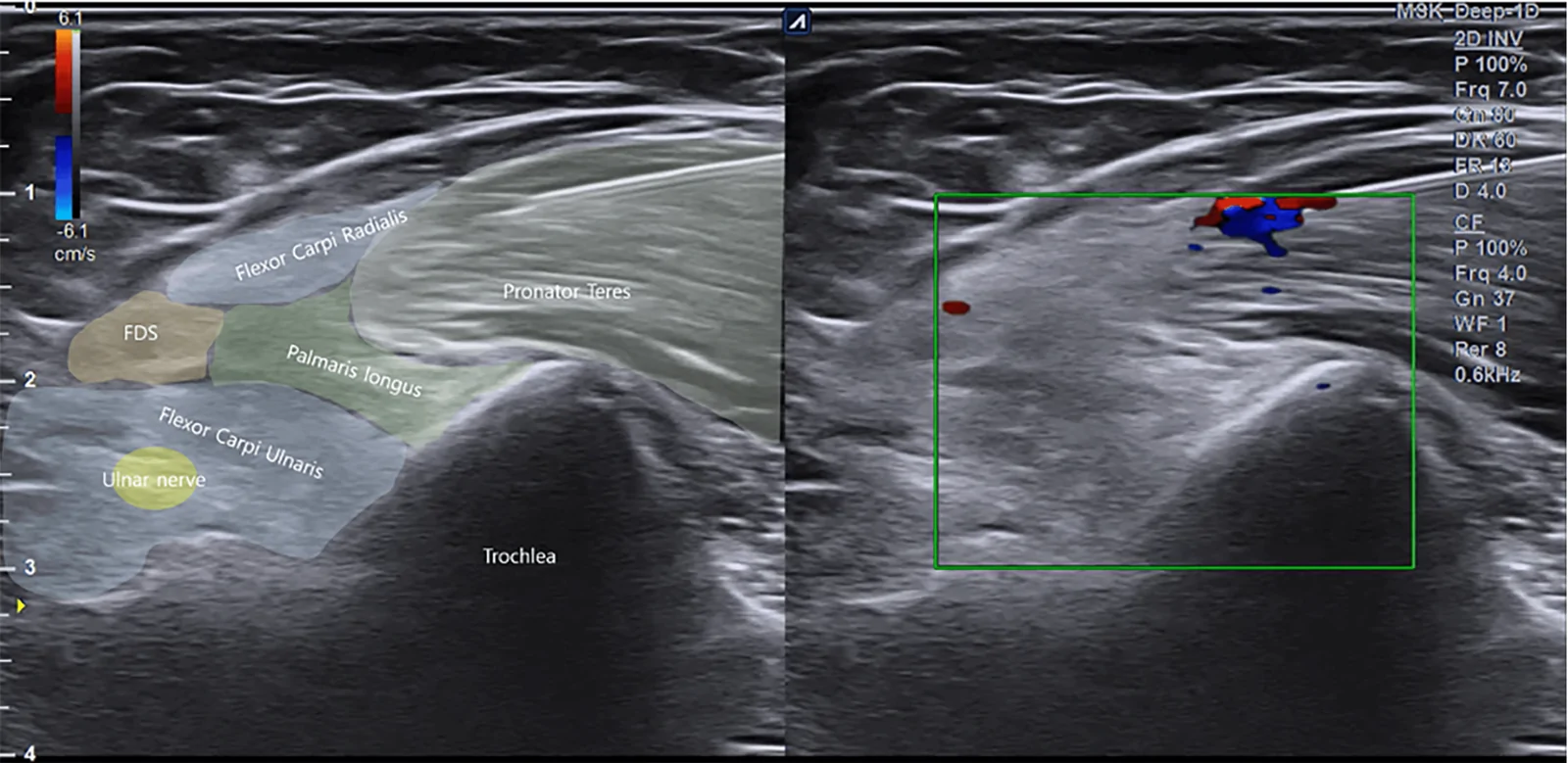
Ultrasound-guided hydrodissection of the symptomatic proximal FDS region Short-axis ultrasound view obtained during ultrasound-guided hydrodissection of the symptomatic proximal FDS region near the medial elbow. A 23-gauge needle was advanced in-plane from lateral to medial under continuous sonographic visualization. Injection of 5% dextrose in water produced separation of the adjacent tissue planes at the targeted region. The procedure was intended to address the observed dynamic gliding abnormality in a minimally invasive manner. FDS, flexor digitorum superficialis Image courtesy: Yonghyun Yoon and King Hei Stanley Lam

After discussion of the treatment options, the patient consented to hydrodissection directed at the site of the dynamic abnormality. Under sterile conditions and real-time ultrasound guidance, a 23-gauge, 6-cm needle was advanced using an in-plane lateral-to-medial approach. The target was the site of abnormal dynamic motion involving the proximal flexor digitorum superficialis and adjacent soft tissue planes near the medial elbow. Given the proximity of the target region to the cubital tunnel, the ulnar nerve was identified sonographically before injection and avoided throughout needle advancement and injection to maintain a safe margin. A volume of 20 mL of 5% dextrose in water was selected to permit adequate hydrostatic separation of adjacent tissue planes under real-time visualization while maintaining patient comfort. No local anesthetic was used. Five percent dextrose in water was selected primarily as a commonly used hydrodissection medium that permits hydrostatic separation of adjacent soft tissue planes without corticosteroid exposure. In this case, it was chosen for its mechanical hydrodissection role rather than to imply a proven proliferative or pathophysiology-modifying effect. The injectate was administered slowly under continuous real-time ultrasound guidance, with each injection pass lasting approximately 60-90 seconds. Injection was paused if the patient reported marked stretching discomfort or if fluid spread beyond the intended tissue plane. The solution was injected under direct visualization, with separation of the tissue planes observed sonographically at the targeted region (Figure 3 and Video 3). The patient tolerated the procedure without immediate complications. Hydrodissection was chosen because it offered a minimally invasive method for addressing a dynamic soft tissue abnormality at the symptomatic site [17-19].

**Video 3:** Real-time ultrasound-guided hydrodissection of the symptomatic proximal FDS region This real-time ultrasound video demonstrates in-plane needle guidance and slow injection of 5% dextrose in water into the symptomatic proximal FDS region. Progressive separation of adjacent tissue planes is seen under direct sonographic visualization. The procedure was performed to target the observed dynamic gliding abnormality while maintaining a safe margin from the nearby ulnar nerve. FDS, flexor digitorum superficialis

Immediately after the procedure, repeat dynamic ultrasound was associated with smoother excursion of the previously abnormal structure and the absence of the previously observed snap or shift at the identified site (Videos 4, 5). The patient reported immediate improvement, with disappearance of the catching sensation during active finger movement (Video 6).

**Video 4:** Post-hydrodissection dynamic long-axis ultrasound showing smoother FDS excursion This long-axis dynamic ultrasound video, obtained immediately after the hydrodissection procedure, shows smoother excursion of the previously abnormal FDS region during active finger motion. Compared with the preprocedure study (Video 1), the irregular, jerky motion is less apparent and was no longer reproduced during the recorded examination. This imaging change was temporally associated with the patient's immediate report of symptom improvement. FDS, flexor digitorum superficialis

**Video 5:** Post-hydrodissection dynamic short-axis ultrasound showing nonreproduction of the previously observed snap or shift This short-axis dynamic ultrasound video, acquired immediately after hydrodissection, demonstrates nonreproduction of the previously observed reproducible snap or shift seen before the procedure (Video 2) during the recorded active finger motion. This postprocedure finding was associated with the patient's immediate subjective improvement, although causality cannot be established from a single case.

**Video 6:** Clinical side-by-side comparison of active finger movement before and after hydrodissection This video provides a direct clinical comparison of the patient's active flexion and extension of the third and fourth digits before (left) and immediately after (right) the hydrodissection procedure. The preprocedure clip shows the characteristic catching and locking. The postprocedure clip shows smoother movement with no obvious triggering during the recorded comparison, corresponding to the patient's immediate subjective improvement.

She was instructed to perform a home program of tendon-gliding exercises and gentle forearm stretching. She was advised to avoid aggravating activities for 48 hours and then gradually resume normal use. Ergonomic counseling was also provided regarding repetitive work tasks, and a night splint was prescribed to maintain the wrist in a neutral position and the metacarpophalangeal joints in slight extension during sleep.

At one week, the patient reported a subjective global improvement of >80% in morning stiffness and only rare, minimal triggering. At two weeks, symptoms were nearly resolved, with only mild muscular discomfort after a full workday. At six weeks, she had complete resolution of pain, stiffness, and triggering and had returned to full occupational duties and recreational rock climbing. Repeat dynamic ultrasound at that time demonstrated persistently normalized motion without recurrence of the previously observed proximal abnormality. No adverse events or delayed complications were identified during follow-up.

The patient stated that the catching sensation improved immediately after the procedure and that, over the following weeks, she was able to return to cooking and rock climbing without the previous locking or morning stiffness.

Written informed consent for publication of the clinical details and media was obtained from the patient.

## Discussion

This case describes an atypical trigger finger presentation in which the expected distal sonographic features of stenosing tenosynovitis were absent, yet a symptom-correlated dynamic abnormality was identified proximally near the medial elbow. The patient's immediate and sustained improvement after targeted hydrodissection suggests that, in selected cases, clinically typical digital triggering may arise from a proximal gliding disorder rather than primary A1 pulley pathology alone.

The conventional trigger finger model remains well supported and clinically useful [1-3]. Standard ultrasound findings typically include A1 pulley thickening, hypoechogenicity, tendon enlargement, tendon sheath fluid, and, in some cases, hypervascularity [10-14]. Most treatment algorithms are therefore appropriately directed at the A1 pulley, whether through conservative care, corticosteroid injection, or surgical release [4-9]. However, the absence of these distal findings in a patient with clear triggering symptoms should prompt consideration of an alternative mechanism rather than premature labeling of the presentation as nonspecific or idiopathic.

Dynamic ultrasonography was central to this case. Unlike static imaging, it allowed direct visualization of the patient's symptomatic motion pattern and localization of the abnormality to the proximal flexor digitorum superficialis. This broader use of ultrasound is consistent with recent literature emphasizing dynamic functional assessment in trigger finger [10,11]. Chuang and McGrouther demonstrated that sonographic evidence of abnormal gliding and adherence-related changes is present in many trigger fingers, supporting the concept that triggering may reflect more than simple pulley stenosis in some patients [15]. Our case extends that concept proximally by showing a motion-dependent abnormality outside the usual distal field of assessment.

At the same time, the mechanism must be interpreted cautiously. While the sonographic abnormality we observed was temporally associated with the patient's triggering, we cannot confirm its histopathological nature. The finding could reflect altered gliding related to connective tissue restriction, fascial laxity, subtle anatomical variation, myofascial dysfunction, or another dynamic mechanical phenomenon. It is also possible that part of the observed sonographic finding was epiphenomenal rather than causal. The term "dynamic instability" in this report therefore refers to a sonographic and functional observation rather than a validated pathological diagnosis. Similarly, the short-axis finding described as a "snap or shift" represents an observed motion abnormality within or adjacent to the tendon; we do not suggest that this constitutes a formal diagnosis of tendon subluxation in the orthopedic sense.

This report does not establish a new disease entity, and the term "proximal trigger finger" should be interpreted, at most, as a descriptive clinical label rather than a validated diagnosis. Nevertheless, the case aligns with the broader literature questioning an exclusively pulley-centric understanding of trigger finger [2,10]. It is also consistent with a recent atypical case report in which triggering was attributed to distal flexor tendinopathy rather than A1 pulley disease [16]. Taken together, these reports suggest that the symptom complex of trigger finger may occasionally reflect pathology at other points along the flexor tendon pathway.

Hydrodissection was selected because the observed abnormality appeared mechanical and gliding-related rather than primarily inflammatory. Hydrodissection has been described as a technique for separating adhered or restricted soft tissue planes around nerves and tendons, usually using saline, anesthetic, corticosteroid-containing solutions, or 5% dextrose in water [17,18]. Prior reports have documented its use in peripheral nerve entrapment syndromes and postoperative flexor tendon adhesions [18,19]. In this case, hydrodissection was associated with immediate sonographic and clinical improvement, suggesting a potential mechanical effect on the observed motion abnormality. However, whether this represented true release of a restricted tissue interface, temporary tissue distension, altered neuromuscular control, or a combination of these mechanisms cannot be determined from a single observation. Although dextrose-containing injectates are sometimes discussed in relation to potential biologic effects, the clinical improvement observed in this case should not be interpreted as evidence of a specific proliferative mechanism. Alternative explanations for the observed improvement should also be considered. Clinical benefit may have reflected temporary tissue distension, altered local neuromechanics, neuromodulatory effects, postprocedural activity modification, placebo response, or a combination of these factors rather than a definitive structural correction alone.

Corticosteroid injection is well established for conventional A1 pulley pathology, where inflammation likely contributes to symptoms [4,7]. However, when the dominant abnormality appears mechanical rather than inflammatory, the rationale for corticosteroid use is less clear. Likewise, in this patient, A1 pulley release would not have addressed the identified proximal abnormality. Because the A1 pulley appeared sonographically normal and the patient improved following treatment directed at the proximal site, distal surgery does not appear to have been necessary in this case. This observation highlights the importance of identifying the specific symptomatic anatomy before recommending surgery [5-9].

This report has several limitations. It describes a single patient, and the prevalence and reproducibility of this pattern are unknown. Interpretation of dynamic ultrasound is operator-dependent. There was no histologic or surgical confirmation of the underlying tissue abnormality. Follow-up was limited to six weeks. Additional limitations include the absence of validated patient-reported outcome measures such as QuickDASH, the lack of blinded ultrasound interpretation, the absence of formal contralateral comparison, and the lack of a standardized quantitative metric for the observed dynamic sonographic abnormality. Although the abnormal motion pattern was reproducibly observed during repeated active flexion-extension cycles, an exact positive cycle count was not prospectively recorded. Accordingly, this case should be viewed as a proof of clinical possibility rather than confirmation of a distinct disease mechanism.

The main lesson from this case is therefore diagnostic rather than mechanistic. In younger patients with occupational overuse, focal proximal tenderness, normal A1 pulley findings, and persistent triggering symptoms, it may be useful to expand ultrasound assessment beyond the distal pulley system and incorporate dynamic examination of the entire flexor pathway.

## Conclusions

This case suggests that clinically typical trigger finger may, in selected patients, be associated with a proximal dynamic flexor tendon abnormality despite normal A1 pulley findings on static ultrasound. In this patient, systematic dynamic ultrasound extending from the hand to the medial elbow localized a symptom-correlated flexor digitorum superficialis motion abnormality near its proximal origin. Targeted hydrodissection was followed by immediate improvement on dynamic imaging and complete short-term clinical resolution. In younger patients with occupational overuse and clinically typical trigger finger but normal A1 pulley findings, extending dynamic ultrasound evaluation proximally may assist in identifying alternative symptomatic sites and guiding targeted treatment in carefully selected patients.
